# Intraoperative and Postoperative Complications Following Open, Laparoscopic, and Hysteroscopic Myomectomies in Saudi Arabia

**DOI:** 10.7759/cureus.7154

**Published:** 2020-03-01

**Authors:** Ahmed A Alharbi, Faisal Alshadadi, Abdullah Alobisi, Abdallah Alsobai, Omar Felimban, Hussain Hudairi, Sultan Ammar, Sultan Alzahrani, Abdullah Abuzaid, Ayman Oraif

**Affiliations:** 1 Urology, King Abdulaziz University, Jeddah, SAU; 2 Surgery, King Abdulaziz University, Jeddah, SAU; 3 Obstetrics and Gynecology, College of Medicine, King Abdulaziz University, Jeddah, SAU; 4 General Surgery, College of Medicine, Jeddah University, Jeddah, SAU; 5 Internal Medicine, King Abdulaziz University Hospital, Jeddah, SAU; 6 Obstetrics and Gynecology, Jeddah University, Jeddah, SAU

**Keywords:** prevalence, complications, myomectomy, laparoscopic

## Abstract

Background

The aim of this study was to broaden our knowledge regarding the complications of myomectomy to better understand how to prevent them from occurring. Another aim was to compare surgical approaches, especially with the current research limitations surrounding this topic in Saudi Arabia.

Methods

This retrospective study was conducted in a cohort of 263 women who underwent surgical myomectomy, without any exclusion criteria. We used our hospital electronic medical records program called Phoenix to obtain all the data regarding clinical presentation, intraoperative findings, intraoperative and postoperative complications, and hospital stay, and then statistically analyzed these findings.

Results

Results were divided depending on the type of surgery. The mean age of open, laparoscopic, and hysteroscopic myomectomy groups were 40.82 years, 42.05 years, and 44.43 years, respectively. There were 213 (80.98%) open, 34 (12.93%) laparoscopic, and 16 (6.09%) hysteroscopic myomectomies. The most common indication in all groups was bleeding. The mean estimated blood loss and duration of surgery for open, laparoscopic, and hysteroscopic myomectomy groups were: 576.13 mL and 103.05 min, 333.21 mL and 56.91 min, and 306.29 mL and 104.19 min, respectively. The total complication rate for each group was 10.8% in open, 2.94% in laparoscopic, and 6.25% in hysteroscopic myomectomies.

Conclusion

Laparoscopy is considered the more effective option for myomectomy than both laparotomy and hysterectomy in terms of surgery duration, hospital stay, and prevalence of complications. However, it is a technically challenging operation that requires experienced surgeons to perform. Based on the information we gathered, we recommend our institute to implement laparoscopy instead of laparotomy myomectomy, which is the current standard procedure in our hospital.

## Introduction

Uterine leiomyomas (UM) of the female reproductive tract are the most common non-malignant tumors that emerge from the smooth muscle cells of the uterus [1-2]. UM is associated with high levels of morbidity, occurring in 25% of women of reproductive age [3-4]. The rate of occurrence of fibroids during pregnancy is 0.05%-5% [5]. Their growth depends on many factors, including hormones, smooth muscle injury, growth factors, and genetic predisposition, and they can be submucosal, subserosal, intramural, or pedunculated [6-7].

One way to treat UM is myomectomy, a surgical procedure for removing myomas. Myomectomy can be carried out through a number of methods: laparotomy, laparoscopy (LM), and hysteroscopy (HM). Laparotomy is also known as open myomectomy (OM) and is considered the most efficient way to relieve symptoms but at the risk of infection and hemorrhage. HM is the gold standard treatment for women of reproductive age that still desire to conceive after the operation [8-10].

Indications for surgical intervention of UM encompass pain or pressure that affects quality of life, heavy uterine bleeding, infertility, suspicion of malignancy, growth following menopause, urinary frequency, and symptoms of obstruction [11]. Fibroids are estimated to be the cause of approximately 7% of recurrent spontaneous abortions [12].

OM is the most efficient method for symptom relief, but it is associated with risks such as infection, hemorrhage, and the need to switch to hysterectomy [13].

A retrospective study carried out in South Korea stated that the majority of fibroids (42.3%) were subserosal [14]. Infertility was the most common indication for surgery. This finding is supported by another study, which also revealed that the incidences of intraoperative and postoperative complications were low (major - 2.62%, minor - 4.54). The major complications were described as hematomas, hemorrhages, and bowel or urinary tract injury; the minor complications included wound infection and cystitis [7]. Furthermore, a study conducted in London in 2012 stated that the most common major complication in abdominal myomectomy is bleeding [15]. Additionally, another study in Taiwan revealed that there was no difference in the rate of complications between older and younger patients [16].

The data concerning the impact of myomas on the general and reproductive health of women remain limited in Saudi Arabia. The goal of this study is to broaden our knowledge regarding the complications of myomectomy to better understand how to treat and prevent them from occurring.

## Materials and methods

Study design

A non-interventional retrospective study

Study setting

The study was conducted through the review of data of patients who underwent surgical myomectomy at King Abdulaziz University Hospital (KAUH) during the period of July 2010 to July 2018. KAUH is one of the biggest tertiary referral and teaching centers in the western region of Saudi Arabia, with a capacity of 800 beds.

Participants and data collection

A retrospective study was conducted in a cohort of 263 women who underwent surgical myomectomy, without any exclusion criteria. We used our hospital electronic medical records program called Phoenix to obtain data. Datasheets were used to collect our information, and the study was divided into two parts. The first part included demographic characteristics, such as age, nationality, gravida, parity, abortion, weight, height, and body mass index (BMI), while the second part contained indications such as bleeding, heavy period, infertility, lower abdominal pain, and accidental sonogram. Also included surgical details were the type, mode, and duration of surgery. Additionally, fibroids' characteristics included type, location, number, length, and width. Also included were complications both during and after surgery, along with the duration of hospital stay after each operation.

Statistical analysis

Data entry was performed using Microsoft Excel 2014, and statistical analysis was performed using SPSS V21 (IBM Corp., Armonk, NY). Categorical variables, including primary variables, nationality, and indications, were described using frequencies. Continuous variables, such as height, weight, and BMI for normally distributed data, were described using mean and standard deviation. Chi-square and Fisher-exact tests were performed to assess the association, odds ratio, and 95% confidence intervals for categorical variables. Pearson χ2 was performed for continuous variables. The association between categorical variables and continuous variables was assessed using an independent t-test, and for multicategory groups, by one-way analysis of variance (ANOVA). A p-value of less than 0.05 was considered significant.

Confidentiality and ethical approval

We removed all identifying variables of patients. Only anonymous data were used to ensure privacy and confidentiality. Additionally, the principal investigator was the only individual with access to all data. The data were disposed of after finishing the study. Approval was obtained from the Institutional Review Board of King Abdulaziz University Hospital.

## Results

**Figure 1:**
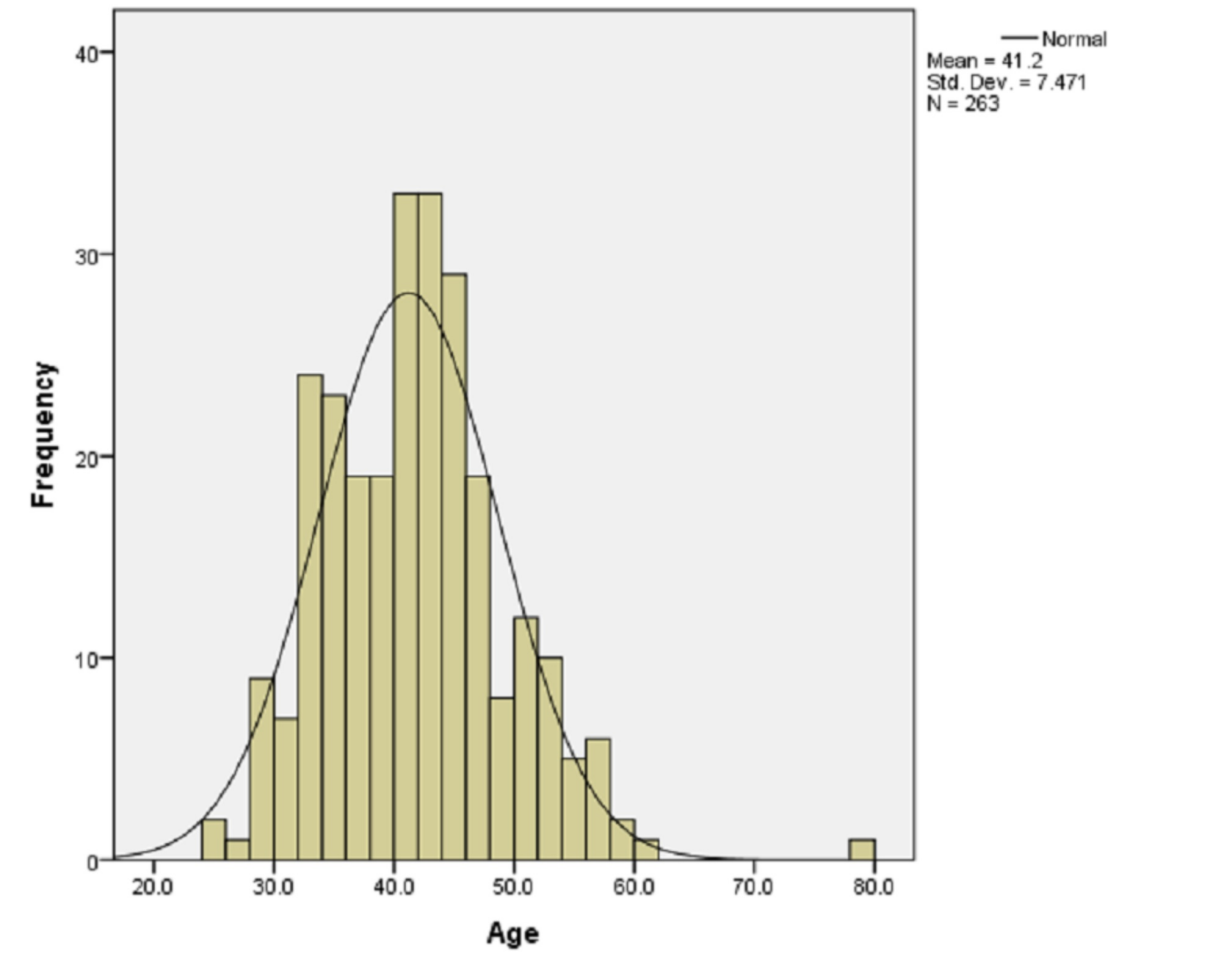
Frequencies of ages in the sample

During the study period, 263 women underwent myomectomy and were included in the analysis. Our demographic study showed that 155 (58.9%) women were Saudi while 108 (41.1%) were non-Saudi. The mean age of the patients was 41.2 ± 7.47 years (Figure 1). Furthermore, the mean weight was 69.38 ± 15.43 kg and the average height was 1.57 ± 0.79 meters. The mean BMI was 28.18 ± 5.97. The average gravida was 1.8 ± 2.3 and para was 1.34 ± 1.82. The mean number of abortions was 0.45 ± 1.16.

The most common indication for myomectomy was bleeding, with 114 cases (43.3%). Patients presenting with menorrhagia accounted for 93 cases (35.4%), accidental sonogram accounted for 90 cases (34.2%), lower abdominal pain accounted for 76 cases (28.9%), infertility accounted for 35 cases (13.3%), dysmenorrhea accounted for 15 cases (5.7%), abortion accounted for 12 cases (4.6%), urinary tract symptoms accounted for 11 cases (4.2%), menometrorrhagia accounted for three cases (1.1%), and constipation accounted for three cases (1.1%).

**Figure 2:**
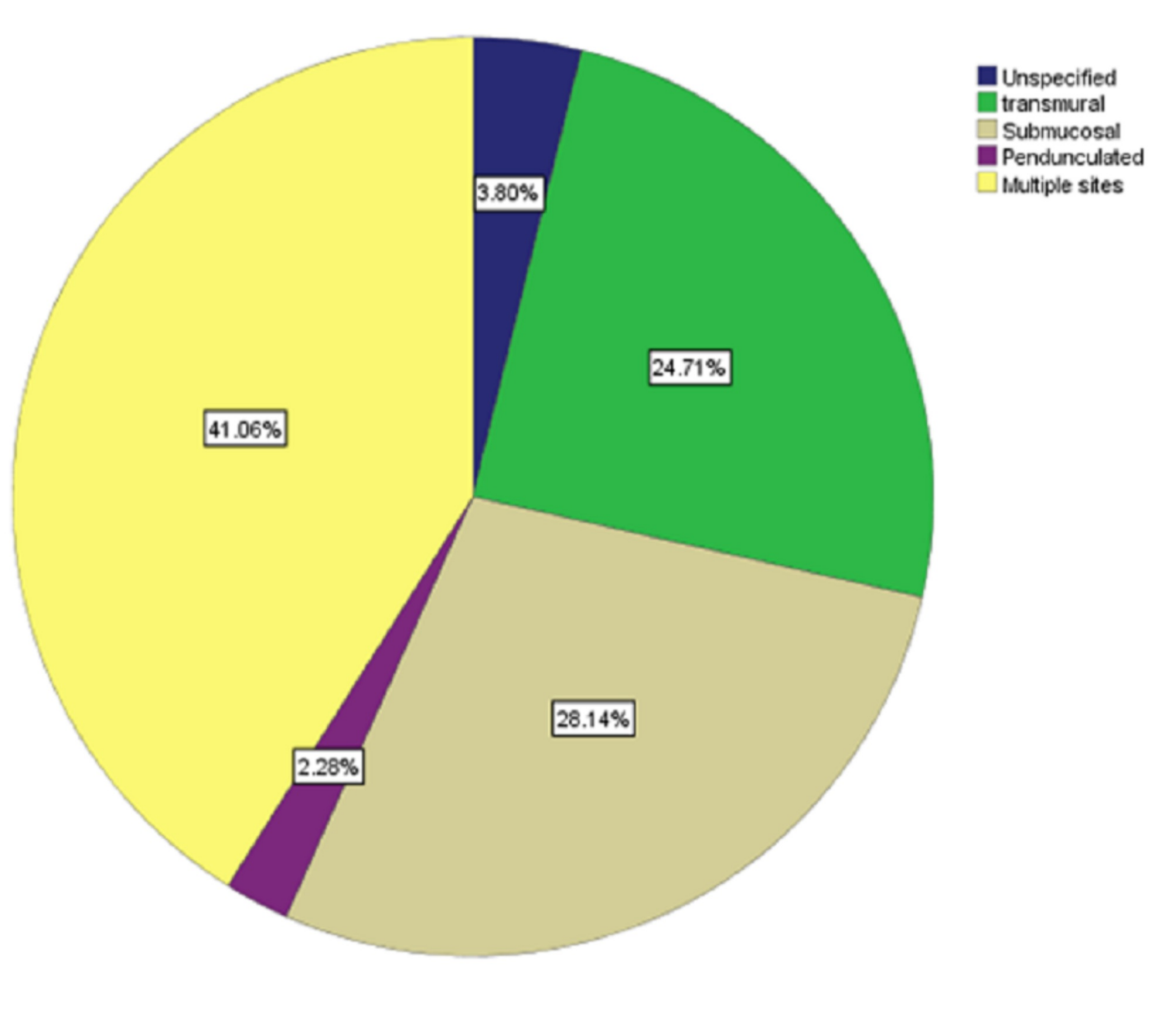
Percentage of each type of fibroid in the sample

The analysis of the detailed surgical outcomes was as follows: the mean number of fibroids was 3.59 ± 3.89. The mean length of fibroids was 7.72 ± 3.3 cm, ranging from 1.20 cm to 20 cm, and the mean (SD) width of the fibroids was 6.97 ± 3.05 cm, ranging from 1 cm to 18 cm. Most fibroids were in multiple sites (108 cases, 41.1%), submucosal (74 cases, 28.1%), transmural (65 cases, 24.7%), unspecified (10 cases, 3.8%), and, less frequently, pedunculated (6 cases, 2.3%) (Figure 2).

The main surgical approach in this study was laparotomy (213 cases, 81%), laparoscopy (34 cases, 12.9%), and hysteroscopy (16 cases, 6.1%). The mode of surgery was elective in 255 cases (97%) and from the emergency room (ER) in eight cases (3%). The mean duration of surgery was 97.15 ± 48.08 min (range 27-330 min). The estimated blood loss was 534.5 ± 445.9 mL (range 40-3000 mL). The average length of hospital stay was 3 ± 2.04 days (range 1-14 days). The mean hemoglobin level before surgery was 11.05 ± 1.88 g/dL, and after surgery, it was 9.41 ± 1.6 g/dL.

The total complication rate was 30 cases (11.4%) in our study. The complication during surgery was 11 cases (4.2%) from the total sample size. There were seven (2.7%) women who had bleeding during surgery, with a mean of estimated blood loss of 1928 ± 534.5 mL. In addition, four (1.5%) women had a perforation in the posterior wall during surgery. There were 25 (9.5%) cases of complications after surgery from the total sample size. The most common complication after surgery was hemorrhage (17 cases, 6.5%), pulmonary embolism (4 cases, 1.5%), urinary tract infections (2 cases, 0.8%), and groin hematoma (2 cases, 0.8%). Also, each of the following conditions had only one case: septic shock, small bowel obstruction, and thrombo-hemorrhagic syndrome. We found six cases (2.3%) of complications during and after surgery. Recurrence of fibroids after mammectomy occurred once in 31 cases (11.18%) and twice in six cases (2.3%).

There was a statistically significant difference, as determined by the independent t-test, between the duration of surgery and 25 (9.51%) patients who had complications after surgery (p < 0.001). Also, the number of fibroids had an impact on the prevalence of the completion of surgery (p < 0.001). There was a moderate positive correlation (r =0.262) between blood loss and duration of surgery. Fisher’s exact test indicated that of 35 women who were infertile, five (14.3%) of them had become pregnant after surgery.

The patients were divided into three groups: those who underwent traditional OM (n = 213), those who underwent LM (n = 34), and those who underwent HM (n = 16). The demographic and clinical characteristics of the three groups are summarized in Table 1. Most of the patients in the OM group were Saudi (130, 61.0%); this also applies to patients in the LM group (18, 52.9%). On the other hand, of the HM group, nine (56.3%) were non-Saudi. The mean age of the patients in the OM group, LM group, and HM group was 40.82 ± 6.94, 42.06 ± 8.72, and 44.44 ± 10.63, respectively. The mean weight was significantly higher in the HM group (74.52 ± 16.21), and there was no significant difference between the OM (68.96 ± 15.12) and LM groups (69.57 ± 17.17). There was no significant difference with regards to the mean height between the three groups. The mean BMI of the patients in the OM group was 27.98 ± 5.87, in the LM group, it was 28.27 ± 6.01, and in the HM group, it was 30.51 ± 7.13.

**Table 1:** Demographics and baseline characteristics of the participating patients (n=263); values are presented as mean ± SD or n (%) ^a ^Data are presented as 95% confidence interval; ^b^ p-values were derived via one-way ANOVA for categorical and continuous variables, and chi-square test for categoric variables BMI: body mass index; ANOVA: analysis of variance

	n	OM group	n	LM group	n	HM group	p-value^b^
Maternal Age (years)	213	40.82 ± 6.94 (39.89 - 41.76)^a^	34	42.06 ± 8.72 (39.02 - 45.10)	16	44.44 ± 10.63 (38.78 - 50.10)	0.135
Height (m)	-	1.57 ± 0.08 (1.56 - 1.58)	-	1.57 ± 0.07 (1.54 - 1.59)	-	1.57 ± 0.09 (1.52 - 1.62)	0.954
Weight (kg)	-	68.96 ± 15.12 (66.79 - 71.12)	-	69.57 ± 17.17 (63.04 - 76.09)	-	74.52 ± 16.21 (65.54-83.50)	0.406
BMI	-	27.98 ± 5.87 (27.14 - 28.82)	-	28.27 ± 6.01 (25.99 - 30.56)	-	30.51 ± 7.13 (26.56 - 34.46)	0.289
Gravida	145	1.77 ± 2.32 (1.38 - 2.15)	27	2.11 ± 2.78 (1.01 - 3.21)	12	1.83 ± 2.08 (0.51 - 3.16)	0.786
Para	146	1.29 ± 1.78 (1.00 - 1.59)	27	1.63 ± 2.22 (0.75 – 2.51)	12	1.25 ± 1.42 (0.35 - 2.15)	0.673
Abortion	145	0.43 ± 1.21 (0.23 - 0.63)	27	0.48 ± 0.94 (0.11 - 0.85)	12	0.58 ± 1.08 (-0.11 - 1.27)	0.892
Nationality							
Saudi	130	(61.0%)	18	(52.9%)	7	(43.8%)	- -
Non-Saudi	83	(39.0%)	16	(47.1%)	9	(56.3%)	

Upon analyzing the data, we found that the most frequent reason for women to undergo all types of myomectomy (OM, LM, and HM) was bleeding. The prevalence we calculated for OM, LM, and HM were: 88 (77.2%), 19 (16.7%), and seven (6.1%), respectively. Bleeding was followed closely by an incidental finding on sonogram as the second most common indication for OM (81, 90%) and HM (5, 5.6%). However, we noted that the second most common indication for LM myomectomy was menorrhagia (14, 15.1%). Additionally, menorrhagia is also the third most common indication for OM (74, 79.6%). In the case of HM, menorrhagia and incidental finding had the same prevalence (5, 5.6%). Furthermore, the third most common indication for LM myomectomy was lower abdominal pain, although the percentage of this indication for OM and HM myomectomy was (86.6%, 3.9%), respectively (Table 2).

**Table 2:** Indications for surgery ^a^p-values were derived via one-way ANOVA for categorical and continuous variables, and chi-square test for categoric variables ANOVA: analysis of variance

Indication	OM group	LM group	HM group	No. (%)	p-value^a^
Bleeding	88(77.2%)	19(16.7%)	7(6.1%)	114(100%)	0.282
Menorrhagia	74(79.6%)	14(15.1%)	5(5.4%)	93(100%)	0.720
Infertility	31(88.6%)	3(8.6%)	1(2.9%)	35(100%)	0.456
Abortion	12(100%)	0(0.0%)	0(0.0%)	12(100%)	0.229
Lower abdominal pain	66(86.8%)	7(9.2%)	3(3.9%)	76(100%)	0.302
Constipation	2(66.7%)	0(0.0%)	1(33.3%)	3(100%)	0.124
Urinary tract symptoms	10(90.9%)	1(9.1%)	0(0.0%)	11(100%)	0.616
Accidental sonogram	81(90.0%)	4(4.4%)	5(5.6%)	90(100%)	0.011
Menometrorrhagia	3(100%)	0(0.0%)	0(0.0%)	3(100%)	0.700
Dysmenorrhea	15(100%)	0(0.0%)	0(0.0%)	15(100%)	0.155

Concerning the mode of surgery, elective surgeries (OM=207, LM=34, and HM=14) were clearly higher than emergency surgeries (OM=6, LM=0, and HM=2) in all groups.

The mean duration of surgery for the OM, LM, and HM groups was: 103.05 ± 48.80, 56.91 ± 17.9 and 104.19 ± 42.35, respectively. Furthermore, blood loss was significantly higher in the OM group (576.13 ± 447.41) and significantly lower in the HM group (306.29 ± 360.49) than in the M group (333.21 ± 392.50). The mean volume of hemoglobin measured one day before the operation for the OM, LM, and HM groups was 11.02 ± 1.86, 11.16 ± 2.14, and 11.21 ± 1.64, respectively. After the surgery, there was no significant change in hemoglobin OM: 9.41 ± 1.61, LM: 9.48 ± 1.85 and HM: 9.40 ± 0.95.

With regards to complications during surgery, the LM group had no complications. On the other hand, six cases of bleeding occurred in the OM group with a mean estimated blood loss of 2000 ± 547.72 and one case in the HM group (Table 3). Additionally, there were only four cases of perforation of the posterior wall, which only occurred in the OM group

**Table 3:** Surgical characteristics and outcomes ^a ^Hemoglobin 1 day before surgery, ^b^ Hemoglobin 1 day after surgery, ^c^ p-values were derived via one-way ANOVA for categorical and continuous variables, and chi-square test for categoric variables UTI: urinary tract infection; EBL: estimated blood loss; ANOVA: analysis of variance

		OM group	LM group	HM group	No. (%)	P value^c^
Number of patients		213	34	16		
Mode of surgery						
Elective		207(81.2%)	34(13.3%)	14(5.5%)	255(100%)	0.05
Emergency		6(75%)	0(0.0%)	2(25%)	8(100%)	
Duration of surgery(min)		103.05 ± 48.80	56.91 ± 17.91	104.19 ± 42.35	-	0.000
EBL (mL)		576.13 ± 447.41	333.21 ± 392.50	306.29 ± 360.49	-	0.003
Hemoglobin preoperative^a^		11.02 ± 1.86	11.16 ± 2.14	11.21 ± 1.64	-	0.875
Hemoglobin postoperative^b^		9.41 ± 1.61	9.48 ± 1.85	9.40 ± 0.95	-	0.982
Complications during surgery						
Perforation of posterior wall		4(100%)	0	0	4(100%)	0.621
Bleeding		6(85.7%)	0	1(14.3%)	7(100%)	0.418
Complications after surgery						
Hemorrhage		17(100%)	0	0	17(100%)	0.118
UTI		2(100%)	0	0	2(100%)	0.789
Groin hematoma		1(50%)	0	1(50%)	2(100%)	0.032
Pulmonary embolism		4(100%)	0	0	4(100%)	0.621
Small bowel obstruction		0	1(100%)	0	1(100%)	0.034
Thrombo-hemorrhagic syndrome		1(100%)	0	0	1(100%)	0.889
Septic shock.		1(100%)	0	0	1(100%)	0.889
Duration of hospital stay (day)		3.58 ± 2.05	1.64 ± 1.41	3.15 ± 1.40	-	0.000

With regard to the type of fibroid, we found that the majority of fibroids were of multiple sites (OM group 84.3%, LM group 9.3%, and HM group 6.5%). This was followed closely by submucosal sites (OM group 63.5%, LM group 28.4%, and HM group 8.1%) and transmural sites (OM group 93.8%, LM group 1.5%, and HM group 4.6%) (Table 4). The mean number of fibroids was as follows: OM group 3.49 ± 3.83, LM group 4.18 ± 4.22, and HM group 3.75 ± 4.01. The mean length of the fibroids was: OM group 7.99 ± 3.35, LM group 5.45 ± 2.14, and HM group 7.26 ± 2.87. The mean width of the fibroid was: OM group 7.18 ± 3.70, LM group 5.32 ± 2.90, and HM group 6.46 ± 2.28) (Table 4).

**Table 4:** Fibroid characteristics ^a^ p-values were derived via one-way ANOVA for categorical and continues variables, and chi-square test for categoric variables ANOVA: analysis of variance

	OM GROUP	LM GROUP	HM GROUP	No. (%)	p-value^a^
Type of fibroid					0.000
Unspecified	10(100%)	0	0	10(100%)	-
Transmural	61(93.8%)	1(1.5%)	3(4.6%)	65(100%)	-
Submucosal	47(63.5%)	21(28.4%)	6(8.1%)	74(100%)	-
Pedunculated	4(66.7%)	2(33.3%)	0	6(100%)	-
Multiple sites	91(84.3%)	10(9.3%)	7(6.5%)	108(100%)	-
Number of fibroids	(3.49±3.83)	(4.18±4.22)	(3.75±4.01)	-	0.628
Length	(7.99±3.35)	(5.45±2.14)	(7.26±2.78)	-	0.005
Width	(7.18±3.70)	(5.32±2.90)	(6.46±2.28)	-	0.035

## Discussion

The aim of this study was to broaden the knowledge regarding the complications of myomectomy, to better understand how to prevent them from occurring. We also aimed to compare surgical approaches, especially with the current research limitations surrounding this topic in Saudi Arabia.

In regard to myomectomy, there are many surgical approaches, including OM. In the current study, we found that the median duration of the surgery is 92 min. Compared to other studies, this was higher in Barakat, Chang, and Sangha, with 126 min, 120 min, and 127.5 min, respectively [17-19]. The time from the incision to closure is defined as actual surgical time. However, this is difficult to compare with other studies because of numerous variables, including patient characteristics, settings, and surgical techniques. Despite this, we believe that the present study found a decreased duration of surgery due to the fact that our institute preforms more open myomectomies than other surgical approaches such as laparoscopy or hysteroscopy procedures. This has led our surgeons to be more skillful and experienced in OM.

On the other hand, we observed that estimated blood loss (EBL) was twice as high compared to the previous studies mentioned [17-18]. Our study demonstrated an EBL with a median of 500 mL as compared to 200 mL in Barakat, 200 mL in Sangha, and 150 mL in Chang [17-19]. The reason for the high value in our study is that one of our patients lost 3000 mL in the procedure, and six procedures also reported a 2000 mL loss, which led to the high values in our analysis.

Regarding complications in OM, out of a total of 213 surgeries, only 23 cases (10.8%) reported complications, while Chang operated 74 with a complication rate of 20 cases (27.0%) [18]. Furthermore, postoperative complications were observed in 26 cases (12.20%) with bleeding being the most common (17, 65%). This is quite high as compared with those in the Barakat and Sangha studies, which only reported one postoperative complication case each [17,19]. These results can be explained by assessing the co-morbidities related to the cases complicated.

The mean age of the participants was comparable with other studies [18-21]. Additionally, our results show that the mean BMI was slightly higher (27.98 ± 5.87) than that in the study by Nash (26.50 ± 6.16) [20]. Our study showed comparable results with other studies in terms of length of hospital stay. The median length of stay was 3.00 days while Barakat, Chang, and Sangha also showed the same results [17-19].

Laparoscopic myomectomy is a recently acknowledged surgical approach for myomectomy. It is a technically demanding procedure, and its success depends on the surgeon’s skill rather than on the number and size of the myoma [7]. In this study, the most common indication for a woman to undergo laparoscopic surgical removal of a myoma was abnormal bleeding while in a study by Paul et al., infertility was the most common indication [7]. The most likely reason is that women in our region who suffer from infertility usually do not seek medical advice at a governmental health care provider due to stigma-related issues in our society.

The average operating time was 56.91 min, which was much lower than the study by Paul et al. (93 min) and Sizzi (107.71 min) [7,22]. The above observations are to be taken in light of the fact that our sample of laparoscopic procedures (n=34) was lower than the studies by Paul et al. (n=1001) and Sizzi (n=2050) [7,22].

Our study found that the average length of hospital stay is comparable with the studies done by Paul et al. and Sizzi but less than the study done by Sangha [7,19,22]. In normal circumstances, part of preoperative counseling is to facilitate a quick return to normal activity. This is achieved through active mobilization using a series of small steps like encouraging the woman to empty her bladder herself. This is done to convince her to go home confidently after surgery.

The total complication rate was 2.94%, with only one case of small bowel obstruction as a postoperative complication. While the study done by Chang showed a much higher rate of 19.8% (16 cases, n=81), a study done by Wang reported no complications (n=28) [18,23]. We believe the reason for the small number of laparoscopic myomectomies reported in our study is the recent introduction of the procedure in our institute and the lack of a number of surgeons who are capable of performing laparoscopy. We believe that in the present future, more cases will be reported. The mean age of our patients (42.06 ± 8.72) was comparable with Sangha, but higher than Paul et al. and Sizzi [7,19,22].

Hysteroscopy is one of the main procedures employed by gynecologists to remove uterine fibroids. It is mostly used for the removal of submucous fibroids. With the advent of hysteroscopic surgery, operative hysteroscopies can manage most intrauterine surgical problems with fast recovery [19].

The most common indication for surgery is abnormal uterine bleeding, which was similar to the study by Polena [24]. This can be due to the fact that, usually, submucosal myomas are an indication of hysteroscopic intervention, which have been implicated more than subserous and intramural tumors as a cause of bleeding, presumably due to the distortion of the cavity and an increase in the bleeding surface of the endometrium.

With regard to the complication rate, a total of two cases (12.5%) were reported out of the 16 operated. Meanwhile, a study done by Wang reported five cases (16.1%) out of 40 operated [25]. The mean operative time and EBL were higher than those reported by Wang [25]. The reason for this is that some of our procedures were combined with additional ones such as colon resection. The mean age of the participants was comparable with that reported by Polena, yet higher than that reported by Wang [24-25].

In this study, we compared the clinical data and surgical outcomes of patients who underwent laparoscopic and hysteroscopic myomectomy. With regard to the indication of surgery, both groups had bleeding as the most common indication. Furthermore, the EBL in both groups was slightly similar. The mean duration of surgery and length of hospital stay were quite a lot higher in the HM group, probably because of the additional procedures that were done during most of the hysteroscopic cases, which lead to the longer operation time and hospital stay.

The complication rate during and after surgery was found to be low in both groups, with only one case reported in the LM group and two cases in the HM group. This could be in light of the improved efficacy and safety of hysteroscopic and laparoscopic myomectomy when compared to the OM group. There was no significant difference between the mean preoperative Hb, postoperative Hb, age, weight, height, and BMI in both groups.

Limitations and recommendations

This study has some limitations; importantly, the sample size is very limited, as we used the Phoenix system to collect patient data. Some data were missing or not updated, and these were deleted from our study. In the future, we recommend performing a multicenter, randomized, clinical trial approach to assess the benefits and drawbacks of each surgical approach.

## Conclusions

Laparoscopy is considered the more effective option for myomectomy than both laparotomy and hysterectomy, in terms of surgery duration, hospital stay, and prevalence of complications. However, it is a technically challenging operation that needs to be performed by experienced surgeons. Based on the information we gathered, we recommend that our institute implement laparoscopy instead of laparotomy myomectomy, which is the current standard procedure in our hospital.

## References

[1] Baird DD, Dunson DB, Hill MC, Cousins D, Schectman JM (2003). High cumulative incidence of uterine leiomyoma in black and white women: ultrasound evidence. Am J Obstet Gynecol.

[2] Kundu S, Iwanuk C, Staboulidou I (2018). Morbidity, fertility and pregnancy outcomes after myoma enucleation by laparoscopy versus laparotomy. Arch Gynecol Obstet.

[3] Pron G, Cohen M, Soucie J, Garvin G, Vanderburgh L, Bell S (2003). The Ontario Uterine Fibroid Embolization Trial. Part 1. Baseline patient characteristics, fibroid burden, and impact on life. Fertil Steril.

[4] Volkers NA, Hehenkamp WJ, Birnie E, Ankum WM, Reekers JA (2007). Uterine artery embolization versus hysterectomy in the treatment of symptomatic uterine fibroids: 2 years’ outcome from the randomized EMMY trial. Am J Obstet Gynecol.

[5] Rasmussen KL, Knudsen HJ (1994). Effect of uterine fibromas on pregnancy. Ugeskr Laeger.

[6] Wallach EE, Vlahos NF, Birnie E, Ankum WM, Reekers JA (2004). Uterine myomas: an overview of development, clinical features, and management. Obstet Gynecol.

[7] Paul GP, Naik SA, Madhu KN, Thomas T (2010). Complications of laparoscopic myomectomy: a single surgeon’s series of 1001 cases. Aust N Z J Obstet Gynaecol.

[8] Radosa JC, Radosa CG, Mavrova R (2016). Postoperative quality of life and sexual function in premenopausal women undergoing laparoscopic myomectomy for symptomatic fibroids: a prospective observational cohort study. PloS One.

[9] Zhang Y, Hua KQ (2014). Patients' age, myoma size, myoma location, and interval between myomectomy and pregnancy may influence the pregnancy rate and live birth rate after myomectomy. J Laparoendosc Adv Surg Tech.

[10] Hurst BS, Matthews ML, Marshburn PB (2005). Laparoscopic myomectomy for symptomatic uterine myomas. Fertil Steril.

[11] Parker WH (2006). Laparoscopic myomectomy and abdominal myomectomy. Clin Obstet Gynecol.

[12] Khaund A, Lumsden MA (2008). Impact of fibroids on reproductive function. Best Pract Res Clin Obstet Gynaecol.

[13] Wallach EE, Schenker JG, Margalioth EJ (1982). Intrauterine adhesions: an updated appraisal. Fertil Steril.

[14] Kim YS, Choi SD, Bae DH (2010). Risk factors for complications in patients undergoing myomectomy at the time of cesarean section. J Obstet Gynaecol Res.

[15] Pundir J, Krishnan N, Siozos A, Uwins C, Kopeika J, Khalaf Y, El-Toukhy T (2013). Peri-operative morbidity associated with abdominal myomectomy for very large fibroid uteri. Eur J Obstet Gynecol Reprod Biol.

[16] Altgassen C, Kuss S, Berger U, Löning M, Diedrich K, Schneider A (2006). Complications in laparoscopic myomectomy. Surg Endosc.

[17] Barakat EE, Bedaiwy MA, Zimberg S, Nutter B, Nosseir M, Falcone T (2011). Robotic-assisted, laparoscopic, and abdominal myomectomy: a comparison of surgical outcomes. Obstet Gynecol.

[18] Chang CC, Chen W (2012). A comparison of surgical outcomes between laparoscopic and open myomectomy in Southern Taiwan. Int J Gynecol Obstet.

[19] Sangha R, Eisenstein DI, George A, Munkarah A, Wegienka G (2010). Surgical outcomes for robotic-assisted laparoscopic myomectomy compared to abdominal myomectomy. J Robot Surg.

[20] Nash K, Feinglass J, Zei C, Lu G, Mengesha B, Lewicky-Gaupp C, Lin A (2012). Robotic-assisted laparoscopic myomectomy versus abdominal myomectomy: a comparative analysis of surgical outcomes and costs. Arch Gynecol Obstet.

[21] Okogbo FO, Ezechi OC, Loto OM, Ezeobi PM (2011). Uterine Leiomyomata in South Western Nigeria: a clinical study of presentations and management outcome. Afr Health Sci.

[22] Sizzi O, Rossetti A, Malzoni M (2007). Italian multicenter study on complications of laparoscopic myomectomy. J Minim Invasive Gynecol.

[23] Wang CJ, Yu HT, Wu PJ, Su H, Wu KY, Han CM (2013). Laparoscopic myomectomy instead of hysteroscopic myomectomy for large submucous fibroids. Gynecol Minim Invasive Ther.

[24] Polena V, Mergui JL, Perrot N, Poncelet C, Barranger E, Uzan S (2007). Long-term results of hysteroscopic myomectomy in 235 patients. Eur J Obstet Gynecol Reprod Biol.

[25] Wang H, Zhao J, Li X, Li P, Lu C, Tian S, Wang ZH (2016). The indication and curative effect of hysteroscopic and laparoscopic myomectomy for type II submucous myomas. BMC Surg.

